# A meta-analysis of the relationship between vaginal microecology, human papillomavirus infection and cervical intraepithelial neoplasia

**DOI:** 10.1186/s13027-019-0243-8

**Published:** 2019-10-26

**Authors:** Yuejuan Liang, Mengjie Chen, Lu Qin, Bing Wan, He Wang

**Affiliations:** The Department of Gynecological of Guangxi Medical University Cancer Hospital, Nanning City, 530021 Guangxi Zhuang Autonomous Region China

**Keywords:** Vaginal microecology, HPV, CIN, Meta-analysis

## Abstract

Microecology is an emerging discipline in recent years. The female reproductive tract is an important microecological region, and its microecological environment can directly affect women’s cervical health. This meta-analysis aimed to analyze the effects of vaginal microecology on Human papillomavirus (HPV) infection and cervical intraepithelial neoplasia (CIN). PubMed and Web of Science were systematically searched for eligible publications from January 2000 to December 2017. Articles were selected on the basis of specific inclusion and exclusion criteria. The design and quality of all studies were evaluated using the Newcastle-Ottawa Scale (NOS). Odds ratios (ORs) with a 95% confidence interval (95% CI) were calculated. Thirteen eligible studies were selected to evaluate the association of vaginal microecology with HPV infection and CIN. The factors related to HPV infection were bacterial vaginosis (BV) (OR 2.57, 95% CI 1.78–3.71, P<0.05), *Candida albicans* (VVC) (OR 0.63, 95% CI 0.49–0.82, *P* < 0.05), Chlamydia trachomatis (CT) (OR 3.16, 95% CI 2.55–3.90, *P* < 0.05), and Ureaplasma urealyticum (UU) (OR 1.35, 95% CI 1.20–1.51, *P* < 0.05). BV was also related to CIN (OR 1.56, 95% CI 1.21–2.00, *P* < 0.05). This meta-analysis of available literature suggested an intimate association of vaginal microecology and HPV infection with CIN. BV, CT and UU were associated to increased HPV infection, VVC was associated to decreased HPV infection, Lactobacillus is not associated to increased HPV infection, BV was associated to increased CIN development risk. Further large-scale studies are needed to confirm our findings.

## Background

Cervical cancer is the second most common malignant tumor among women worldwide, second only to breast cancer [1]. Human papillomavirus (HPV) infection, especially high-risk human papilloma virus (HR-HPV) persistent infection, is a prerequisite for the development of cervical intraepithelial neoplasia (CIN) and cervical cancer [2]. The HPV infection rate of Chinese women was 15.71% in the previous years, and 84.6% of sexually active women were infected with HPV at least once in their lifetime, but few HPV infections persist and progress to cervical cancer [3]. Over 90% HPV and CIN I, half of CIN II, and 30% of CIN III can be self-contained [4–6]. In most of the cases, the HPV infection is cleared by the immune system within 2 years [7]. The long-term retention of HPV that cannot be removed due to various factors such as cervicitis, multiple sexual partners, smoking, etc. causes the HPV viral load to remain at a high level in the body leading to cervical lesions, and are also closely related to the severity of cervical lesions [8, 9]. Although HPV vaccines can significantly reduce HPV infection rates, most of developing countries are slow to introduce vaccinations [10]. Therefore, one of the primary means of preventing cervical lesions is to detect and block the sustained HR-HPV infection in a timely manner.

Identifying risk factors for the development of CIN and cervical cancer has been the objective of recent studies [11]. Normal vaginal microecology plays an indispensable role in the prevention of female genital tract infection and its alteration is inextricably linked to cervical lesions development [12]. The vagina is mainly composed of its anatomical structure, micro-ecological flora, local immunological and endocrine factors [13]. Although the normal microflora is the core focus of vaginal microecology research, microbes such as lactobacillus, bifidobacteria and bacteroides are mutually constrained and coordinated with the host and the environment to maintain the dynamic balance of the vaginal microecological system [14]. If the vaginal microecological flora loses this dynamic balance and the immune system is impaired, it is easier for foreign microorganisms to invade and cause inflammation within the reproductive tract [15]. The presence of inflammatory stimuli increases the risk of cancer [16]. Studies have shown that genital tract inflammation caused by HPV infection is closely related to tumorigenesis [17]. Studies have also found that the HPV-positive women have a greater diversity of vaginal microbial species than do HPV-negative women; in addition, the microbiome plays an important role in the development of cancer [18, 19]. According to different studies, the vaginal microecology plays a crucial part in preventing HPV infection and accelerating HPV virus clearance and its homeostatic imbalance may be a synergistic factor for HPV infection [20, 21]. The aim of this study was to analyze the relationship between the vaginal environment and HPV or CIN to provide some basis for regulating vaginal microecological balance, blocking HPV infection and intervening in the progression of cervical lesions.

## Materials and methods

### Literature search

Relevant studies on the association between vaginal microecology and HPV or CIN were identified through an extensive search of PubMed and Web of Science between January 2000 and December 2017 based on the following keywords: ‘vaginal microecology’,‘vaginal microenvironment’, ‘vaginal environment’, ‘vaginal microbiota’, ‘vaginal flora’, ‘cervicovaginal coinfections’ or ‘cervical inflammation’, in combination with ‘cervical intraepithelial neoplasia’, ‘cervical lesions’, ‘cervical dysplasia’, ‘human papillomavirus’, ‘papillomavirus infections’ or ‘HPV’. Studies were limited to those written in English. We performed a second search based on the references in the original literature.

### Research selection and data extraction

Studies describing the relationship between vaginal microecology and CIN or HPV infection were included in this meta-analysis. Eligible studies were required to have clinical or pathological diagnostic information related to HPV and CIN. Articles were included if they presented data for calculation. Conference abstracts and other unpublished articles were excluded as these could not be systematically reviewed and the data could not be verified. Duplicate reports with similar content from the same author were excluded. Studies containing special occupational groups were excluded. The selected studies focused mainly on bacterial vaginosis (BV),Trichomonas vaginitis (TV),Chlamydia trachomatis (CT),Ureaplasma urealyticum (UU), lactobacillus, *Candida albicans* (VVC). The design and quality of all studies were evaluated using the Newcastle-Ottawa Scale (NOS) [22]. For each study, the following data were extracted: first author, year of publication, study type, type, number of case, number of control, risk factors and quality.

### Statistical analysis

Data from each observation in the experimental and control groups were extracted. We used Review Manager 5.3 software to analyze the data and calculate the odds ratio (OR) and its 95% confidence interval (95% CI). The results were visualized in a forest plot. Study homogeneity was quantified by I^2^ statistic test. The fixed effect model (Mantel and Haenszel method) was selected if the results showed *P* > 0.10 and I^2^ < 50%; otherwise, the random effect model (DerSimonian and Laird method) was chosen. *P* < 0.05 was considered to be statistically significant.

## Results

We selected 13 studies from hundreds of articles based on established standards. These publications included nine studies on vaginal microecology and HPV infection, three studies on vaginal microecology and CIN and one study on vaginal microecology with HPV infection and CIN. A total of 5639 women were included in the in case group and 19,561 women in the control group.

### Vaginal microecology - cervical human papillomavirus association

#### BV with HPV infection

**Fig. 1:**
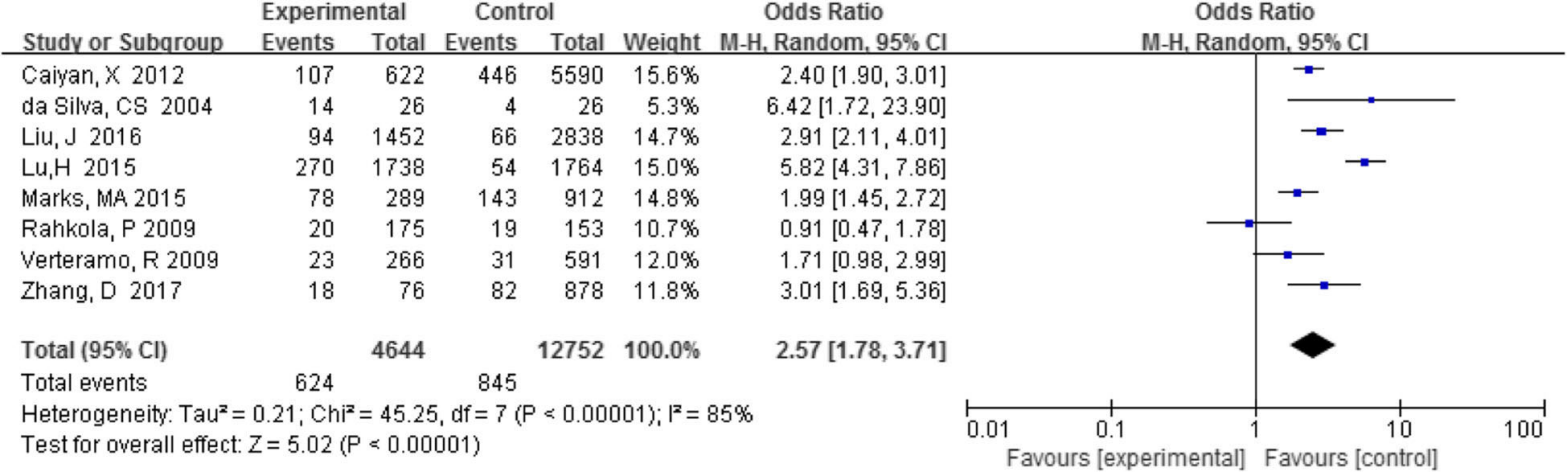
Comparison of BV in the HPV-positive and -negative groups

Eight studies reported a comparison of BV detection rates in HPV-positive and –negative individuals. Analysis of the association between BV and cervical HPV infection showed that HPV prevalence was higher in BV-positive women in seven out of eight studies compared with women without BV. Among these women, the detection rate of BV in the HPV-positive group was 13.4% (624/4644), and that in the HPV-negative group was 6.6% (845/12752). The total results based on all eight studies (OR 2.62, 95% CI 1.84–3.73, *P* < 0.05) were statistically significant (Fig. 1), indicating a positive association between BV and cervical HPV infection.

**Table 1:** Characteristics of the selected studies included in the meta-analysis

Authors	Year of publication	Study type	Type	Number of case	Number of control	risk factors	Quality
da Silva et al. [23]	2004	Case-control	HPV	26	26	bh	7
Gao et al. [24]	2013	Case-control	HPV	32	38	g	7
Lu et al. [25]	2015	Case-control	HPV	1738	1764	bd	6
Caiyan et al. [26]	2012	Case-control	CIN/HPV	374/622	5985/5590	bch	7
Marks et al. [27]	2015	Case-control	HPV	289	912	bd	7
Behbakht et al. [28]	2002	Case-control	CIN	17	34	b	6
Liu et al. [29]	2016	Case-control	HPV	1452	2838	bcde	7
Murta et al. [30]	2000	Case-control	HPV	390	396	ch	7
Rahkola et al. [31]	2009	Case-control	HPV	175	153	b	7
Zhang et al. [32]^a^	2017	Case-control	HPV	76	878	bcdeh	6
Schiff et al. [33]	2000	Case-control	CIN	112	326	bcd	7
Verteramo et al. [34]	2009	Case-control	HPV	266	591	bcdeh	7
Barcelos et al. [35]	2011	Case-control	CIN	70	30	bch	7

Note:b (BV), c (TV), d (CT), e (UU), g (Lactobacillus), h (VVC). ^a^a one participant in 954 women with the laboratory results of vaginal swab specimens missed the data of Candida, CT and UU

#### VVC with HPV infection

**Fig. 2:**
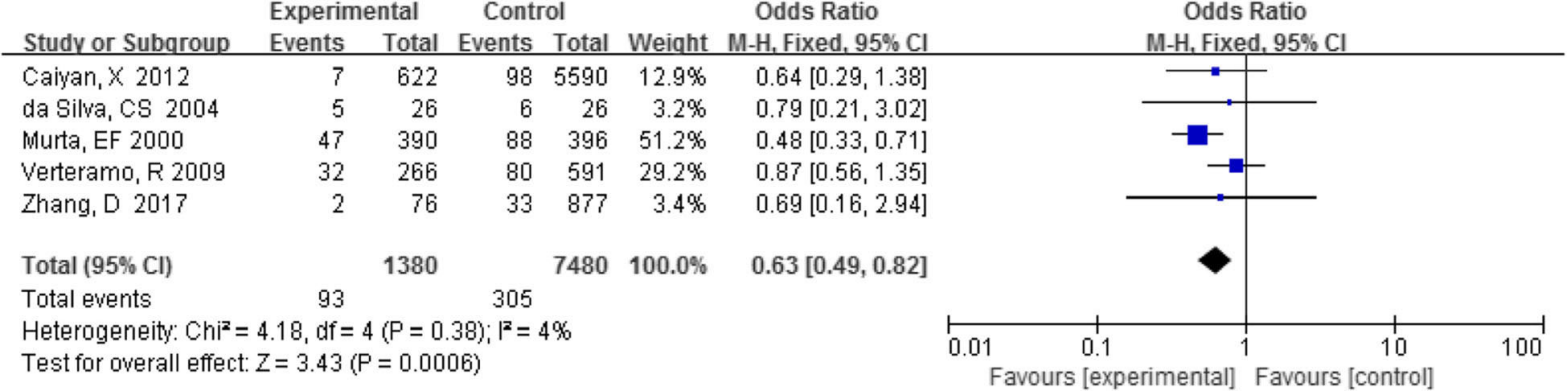
Comparison of VVC in the HPV-positive and -negative groups

Five studies analyzed the association between VVC and HPV infection. The detection rate of VVC in the HPV-positive group was 6.7% (93/1380), and that in the HPV-negative group was 4.1% (305/7480). A summary of all data showed that this association was statistically significant (OR 0.63, 95%CI 0.49–0.82, *P* < 0.05)(Fig. 2).

#### TV with HPV infection

**Fig. 3:**
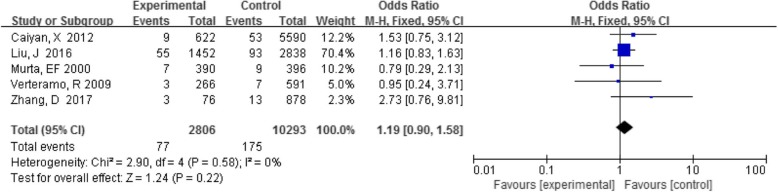
Comparison of TV in the HPV-positive and -negative groups

Five studies analyzed the association between TV and HPV infection, and individual studies showed no association between these infections. The detection rate of TV in the HPV-positive group was 2.7% (77/2806), and that in the HPV-negative group was 1.7% (175/10293). A summary analysis of five studies showed that the OR of Trichomonas did not differ between the HPV-positive and -negative group (OR 1.19, 95% CI 0.90–1.58, *P* = 0.22) (Fig. 3).

#### CT with HPV infection

**Fig. 4:**
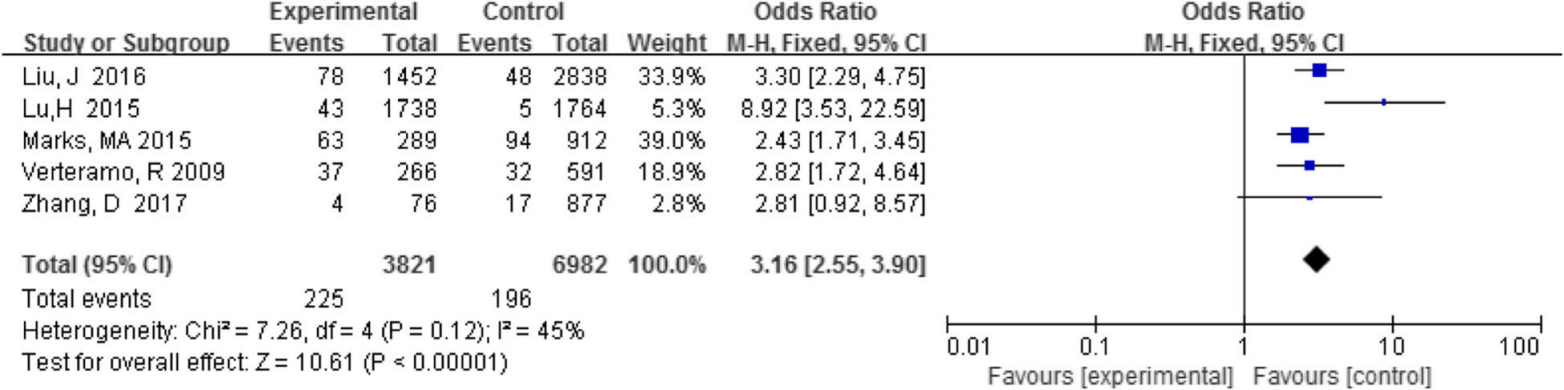
Comparison of CT in the HPV-positive and -negative groups

Five studies analyzed the association between CT and HPV infection. The detection rate of Chlamydia trachomatis was 5.9% (225/3821) in the HPV-positive group and 2.8% (196/6982) in the HPV-negative group. There was a statistically significant association when the results of all studies were analyzed together (OR 3.16, 95% CI 2.55–3.90, *P* < 0.05) (Fig. 4).

#### UU with HPV infection

**Fig. 5:**
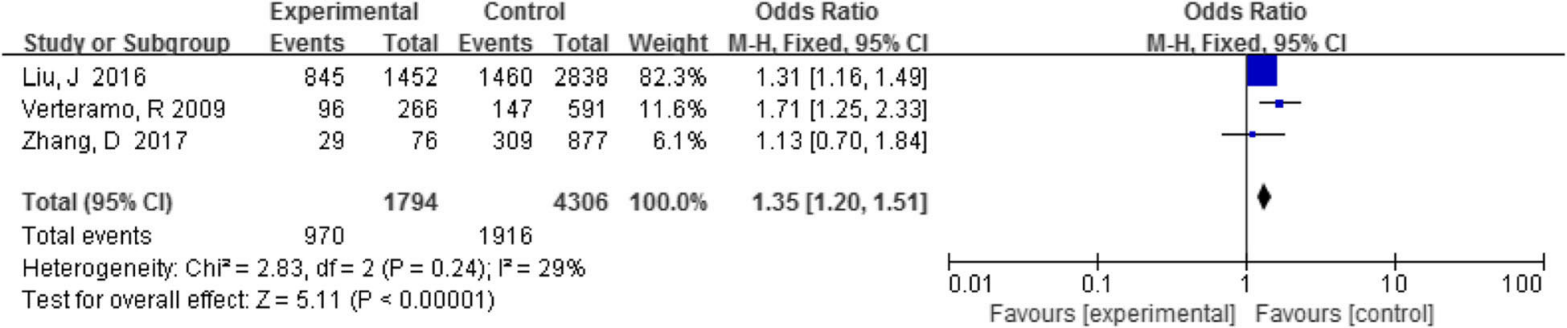
Comparison of UU in the HPV-positive and -negative groups

Three articles analyzed the relationship between UU and HPV infection, and the results of the individual studies were different. There was a statistically significant association when the results of all studies were analyzed together (OR 1.35, 95% CI 1.20–1.51, *P* < 0.05) (Fig. 5). Lu,H [25] also reported that the detection rate of mycoplasma in the HPV-positive group (6.5%) was significantly higher than the HPV-negative group (1.2%).

#### Lactobacillus with HPV infection

The relationship between lactobacilli and HPV infection was studied by a single literature. Gao, W [24] reported that the detection rate of Lactobacillus in HPV-positive group (56.3%) was lower than the HPV-negative group (60.5%).

### Vaginal microecology – CIN association

#### BV with CIN

**Fig. 6:**
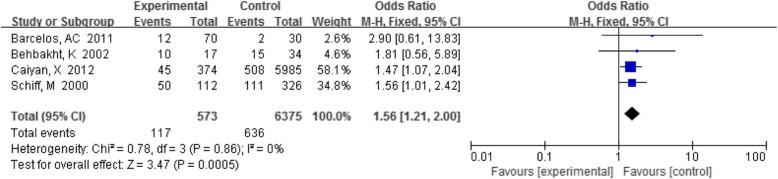
Comparison of BV in the CIN group and control group

Four reports analyzed the correlation between BV and CIN, and the results of a single study were inconsistent. The detection rate of BV in the CIN group was 20.4% (117/573), and in the control group it was 10.0% (636/6375). The summary results showed that the difference was statistically significant (OR 1.56, 95% CI 1.21–2.00, *P* < 0.05) (Fig. 6).

#### TV with CIN

**Fig. 7:**
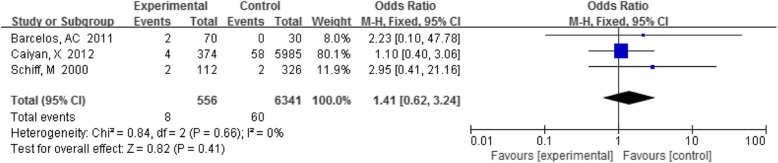
Comparison of TV in the CIN group and control group

Three papers researched the correlation between TV and CIN. All studies concluded that there was no significant correlation between TV and CIN. Among these studies, the detection rate of TV in the CIN group was 1.4% (8/556) and that in the control group was 0.9% (60/6341). As shown in Fig. 7, the summary results revealed that the difference was not statistically significant (OR 1.41, 95% CI 0.62–3.24, *P* = 0.41).

#### VVC with CIN

**Fig. 8:**
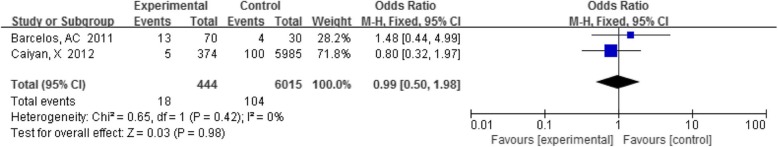
Comparison of VVC in the CIN group and control group

Two papers researched the correlation between Candida infection and CIN and reached the same conclusion that Candida infection has nothing to do with CIN. The detection rate of VVC in the CIN group was 4.1% (18/444), and that in the control group was 1.7% (104/6015). We combined the results to show that VVC and CIN were not significantly associated (OR 0.99, 95% CI 0.50–1.98, *P* = 0.98) (Fig. 8).

## Conclusions

Cervical cancer is the most common malignant tumor in the female reproductive tract [36]. It is well known that infection with oncogenic HPV,especially HR-HPV,is the main etiological agent in the development of CIN and cervical cancer [37]. It is unknown why HR-HPV infection is cancerous in some women whereas in others it is eradicated. There may be individual differences in immunological defense, but local cervical factors may determine the outcome of HPV infection and CIN. With in-depth study of the human microecological system, the role of microecological balance in maintaining human health has been further emphasized [38]. Whether vaginal flora plays a role in persistent HPV infection and the development of CIN has been considered, as vaginal flora is an important factor in the stability of the vaginal environment. In this analysis, we included 13 studies and concluded that BV is associated with HPV infection and CIN, whereas CT and UU are associated with HPV infection.

BV is the most common vaginal infection in women of childbearing age. Previous systematic reviews [39] reported a positive association between BV and cervical HPV infection (OR 1.43, 95% CI 1.11~1.84), which indicated that the presence of BV increases the risk of cervical HPV infection. The mechanism may be an increase in the mucin-degrading enzyme in the vaginal fluid of BV-positive patients, which can promote virulence by disrupting the protective mucosal barrier with the result of increasing the susceptibility to cervical HPV infection by promoting viral adhesion, invasion and eventual integration of HPV genome. It is also possible that the anaerobic bacterial metabolism of BV produces ammonia and carcinogenic ammonia nitrite in vaginal secretions, causing abnormal changes in cervical epithelial cells (such as cervical epithelial cell transformation, exfoliation). This study incorporated new publications to evaluate the relationship between BV and HPV infection, reaching a consistent conclusion that BV is associated with HPV infection. Furthermore, we also found that BV was associated with CIN (OR 1.56, 95% CI 1.21–2.00, *P* < 0.05), therefore BV may increase the risk of cervical HPV infection and CIN development. Sodhani P et al. [40] found that precancerous lesions were more frequently detected in smears of bacterial vaginosis from a total of 24,565 smears (*P* < 0.0001). Meanwhile, Zhang,H et al. [41] reported the cervical microbial diversity was reduced in CIN II/III patients after the loop electrosurgical excision procedure (LEEP). However, we have not been able to prove that curing BV is conducive to the clearance of HPV and the outcome of CIN from this study, and further research is needed.

TV and VVC infections are common genital tract infections in women. *Candida albicans* is an opportunistic pathogen that usually does not cause disease on the vaginal mucosa but may cause disease when the body’s immunity is reduced or the vaginal acidic environment changes [42]. TV is a contagious parasitic disease mainly transmitted through sexual intercourse [43]. Whether the natural history of HPV infection is affected by VVC or TV infection has not been fully investigated. In this meta-analysis, Caiyan,X et al. [26] concluded that TV infection was not associated with HPV and CIN. However, according to recent reports, Feng RM et al. [44] analyzed 25,054 women in rural China using liquid-based cytology and found that HPV was more prevalent in TV-positive women (OR 1.31, 95% CI: 1.11–1.56). However, TV-positive women had a reduced risk of CIN2+, especially among women with HR-HPV infection. Regarding the relationship between VVC and HPV infection or CIN, this meta-analysis found that there was no significant correlation between TV and HPV infection and CIN (*P* > 0.05), and that VVC is a protective factor for HPV infection (OR 0.63,95%CI 0.49–0.82,*P* < 0.05) and had no correlation with CIN(*P* > 0.05). Meanwhile, Engberts MK et al. [45] also concluded that Candida did not increase the risk of developing cervical cancer, and it was reported that Candida could be used as a new adjuvant for HPV therapeutic vaccines as it could enhance the immune response by enhancing T cell proliferation [46]. Based on these results, further research should be conducted to assess the relationship and mechanism between TV or VVC and HPV or CIN to increase patient benefit.

In recent years, reports on the relationship between mycoplasma and chlamydia infection and HPV infection and cervical lesions have gradually increased [47]. Whether mycoplasma and chlamydia infection are synergistic factors in the development of cervical cancer is still controversial in current research. This study found that the detection rate of CT in HPV positive group was 5.9% (225/3821), UU was 5.4% (970/1794), and the detection rate of CT in HPV negative group was 2.8% (196/6982),UU was 4.5% (1916/4305). The differences were statistically significant (OR 3.16,95% CI 2.55–3.90, *P* < 0.05; OR 1.35, 95% CI 1.20–1.51, *P* < 0.05). This indicating that both UU and CT increased the risk of HPV infection; the risk of HPV infection increased by 1.35-times in UU-positive patients and by 3.16-times in CT-positive patients. Different studies have reported that UU plays an important role in initiating abnormalities and persistence of viral cells, and it is a cofactor for HPV to promote precancerous lesions that lead to cervical cancer [48, 49]. The possible mechanism of the association between UU infection and abnormal cervical cytopathology might be related to the combination of several complex infection-associated infammatory responses [50], involving production of reactive oxidative metabolites, increased expression of cytokines, chemokines,and growth and angiogenic factors, decreased cell-mediated immunity, and the generation of free radicals [51]. Valadan M et al. [52] also found a significant association between CT infection and CIN in a case-control study (OR = 5.5, 95% CI 2.4–12.4). The possible mechanism for this association is that chlamydia adsorbs to the genital mucosa after infection, causing damage and inflammatory reactions in genital mucosal epithelial cells, reducing cervical and vaginal immune barriers, and facilitating HPV infection to trigger CIN and cervical cancer [34]. In this meta-analysis, CT and UU were positively associated with HPV infection. However, larger samples and long-term follow-up studies are necessary to further confirm these results.

The changes in vaginal pH may play a major role during the progression of HPV infection and CIN to cervical cancer. Under normal circumstances, Lactobacillus is the most dominant bacterial genus in the female vagina, and it can regulate the structure of vaginal flora and maintain the stability of vaginal microenvironment by producing a variety of bacteriostatic and bacteriocidal metabolites, such as lactic acid, H_2_O_2_, and biosurfactants bacteriocin [13]. HPV infection results in the loss of local lactic acid bacteria, destroying the biological barrier of the local vaginal immune microenvironment, aggravating the destruction of the vaginal environment, promoting the abnormal adhesion of HPV in the vagina, causing a local microecological imbalance in the vagina and destroying the local immune function of the cervix while simultaneously increasing the adhesion, invasion and colonization of abnormal flora [53]. This will form a vicious cycle in the vaginal environment, resulting in the further development of HPV infection, thereby inducing cervical lesions. Clarke MA et a1 [54]. conducted a study of the relationship between vaginal pH and HPV infection in 9165 women and showed that vaginal pH was closely related to HPV infection, especially in women under 35 years of age. In addition, increased vaginal pH in women < 35 years old and > 65 years old increased the risk of multiple HPV infections, indicating the importance of vaginal pH in maintaining the balance of the vaginal environment. In this study, only a single article [24] reported the relationship between HPV infection and Lactobacillus, and the detection rate of Lactobacillus in the HPV-positive group (56.3%) was lower than that in the HPV-negative group (60.5%). Therefore, a large number of samples is needed to verify the relationship between vaginal pH imbalance caused by lactobacillus changes and the development of HPV infection and cervical cancer.

To conclude, this meta-analysis indicated that BV, UU, CT and reduction of Lactobacilli are associated to increase risk of HPV infection and CIN development, while TV and *Candida albicans* infections are not significantly associated to HPV infection and CIN development and may have a protective effect. In short, the female genital tract system is a complex microbial environment, and avoiding a vaginal flora imbalance may have a significant effect on preventing HPV infection and CIN development. This meta-analysis suggests an intimate connection between vaginal microecology and HPV infection or CIN. Considering that these conditions are very common among women worldwide, further research in this field is imperative.

This meta-analysis was limited to that of published studies, which could have caused publication bias, resulting from tendency to selectively publish results that are statistically significant. Additionally, the lack of some literature data may lead to a bias in results. Therefore, more rigorous controlled studies with increased sample sizes are required to provide a more reliable experimental basis.

## Data Availability

All data generated or analyzed during this study are included in this published article.
