# Correction to: A meta-analysis of the relationship between vaginal microecology, human papillomavirus infection and cervical intraepithelial neoplasia

**DOI:** 10.1186/s13027-019-0258-1

**Published:** 2019-12-09

**Authors:** Yuejuan Liang, Mengjie Chen, Lu Qin, Bing Wan, He Wang

**Affiliations:** The Department of Gynecological of Guangxi Medical University Cancer Hospital, Guangxi Zhuang Autonomous Region, Nanning City, 530021 China

Correction to: Infect Agents Cancer (2019) 14: 29

https://doi.org/10.1186/s13027-019-0243-8

In the original publication of this article [1] there was an error in the results section of the article.

The result section showed the following:

- The total results based on all eight studies (OR 2.62, 95% CI 1.84–3.73, P < 0.05) were statistically significant (Fig. 1)

However, the correct information is:

- The total results based on all eight studies (**OR 2.57, 95% CI 1.78–3.71**, P < 0.05) were statistically significant (Fig. 1)

The updated information is shown in bold. The correct information is already available in Fig. 1 of the original publication.
